# On valorization of solvent extracts of *Terminalia arjuna* (arjuna) upon DNA scission and free radical scavenging improves coupling responses and cognitive functions under in vitro conditions

**DOI:** 10.1038/s41598-021-88710-w

**Published:** 2021-05-20

**Authors:** D. K. Meena, A. K. Sahoo, P. P. Srivastava, N. P. Sahu, M. Jadhav, M. Gandhi, H. S. Swain, S. Borah, B. K. Das

**Affiliations:** 1 ICAR-Central Inland Fisheries Research Institute, Barrackpore, Kolkata, 700120 India; 2 ICAR-Central Institute of Fisheries Education, Mumbai, 400061 India; 3 Centre for Nanotechnology and Science, IIT, Mumbai, 400076 India

**Keywords:** Medicinal chemistry, Regenerative medicine

## Abstract

Chronic diseases have been treated using the phytochemical concepts of ethnomedicinal plant-derived herbal products. *Terminalia arjuna*, a significant ethnomedicinal plant, was revisited and reconnoitred for antioxidant, free radical scavenging, and DNA nicking inhibiting activity under H_2O2_ conditions using 21 solvent extracts. Ferric reducing antioxidant power (FRAP), 2,2-diphenyl-1-picrylhydrazyl (DPPH), 2,2′-azino-bis-3-ethylbenzothiazoline-6-sulphonic acid, and nitrous oxide scavenging (%) were found to have a strong positive association and interaction (PCA 1 explains 84.54% variation) with ethanol bark (Etoh-AB) (Meoh-AF). TPC (144.67–1794 µg/mL GAE) and TFC (2.5–34 µM Fe(II)/g were highest in Etoh-AB. In a pattern of combined solvent extracts, Etoh-AB had the highest antioxidant capacity, accompanied by Etoh-AL ≥ Meoh-AB ≥ Dw-AF. With *R*^2^ = 0.94, the DNA nicking inhibition behaviour parameters relative front, relative quantity, band (%), and lane (%) formed a positive significant (*p* < 0.01) connection. For the first time, we show that Etoh-AB nicks supercoiled, circular plasmid DNA in a way that is comparable to normal antioxidants. Normal antioxidants with the ability to prevent DNA nicking include Butylated hydroxy anisole < Butylated hydroxy toluene < ascorbic acid < and Gallic acid. Gallic acid (*m/z* 170.0208 g/mol) and Ellagic acid (*m/z* 302.0063 g/mol were present in high concentrations in solvent extracts. 0.48 mg was found to be the effective concentration for inhibiting relative DNA nicking. The current study is the first of its kind to show that steroid concentrations are higher in bark fractions of acetone, ethanol, and methanol. Furthermore, *T. arjuna* solvent extracts provide a wealth of information on phytochemical profiling, antioxidant ability, and DNA nicking inhibition, which may be useful for exploring the natural way and further research to develop a remedy against geriatric chronic disease. Despite the fact that ethanol is very close to methanol in terms of solvent toxicity, the current study identified it as the preferred solvent. Thus, the current research revisits previous studies and explores the potentiality of non-polar and polar aprotic and polar protic solvent systems, which lend credence to bioactive compounds that may be useful in isolating and formulating safe and cost effective herbal medicament for livestocks and aquaculture, and drugs for deoxygenerative human diseases, and can also be investigated further to instil environmental frugality.

## Introduction

Oxidation is a natural process that results in the formation of free radicals known as Reactive Oxygen Species (ROS) and Reactive Nitrogen Species (RNS) through a series of intermediate by-products^1^. These free radicals play critical roles in the mechanism of cell signalling, while in the case of hyper oxidation, basic cellular biomolecules such as proteins, enzymes, DNA, RNA, lipids, and carbohydrates are thought to be negatively affected by oxidative alteration, resulting in a functional imbalance between free radicals and antioxidants. When these two factors are out of control, severe DNA damage occurs, and multiple degenerative diseases mediated by oxidative stress are responsible for causing lethal and serious anarchy such as cancer, strokes, and ageing^2^. Natural DNA-damaging agents (genotoxins) such as UV light, dietary factors, and free radicals are continuously in contact with animals. Damaged DNA begins to accumulate in major functional and metabolic organs (brain, muscle, liver, kidney, and stem cells), resulting in ageing, gastric ulcer, carcinogenesis, neurodegenerative diseases, inflammation, gene expression decrease, and functional ability loss^3,4^. DNA damage is also linked to oxidative stress, which leads to arteriosclerosis. It has been stated that using a leaf extract of *Launaea taraxacifolia* to inhibit DNA damage nicking could reverse arteriosclerosis^5^. TPC and antioxidant studies showed that phenolics are the most bioactive compounds for *T. bellerica's* antioxidant, DNA defensive, and antibacterial activities^6^. On the other hand, some cancer therapeutic agents destroy the normal ceel, resulting in DNA damage. It is critical to test the combination of DNA damaging and DNA repair agents in order to improve the chances of destroying cancer cells and reducing damage to normal tissues by administering them selectively to the cancer cell^7^. DNA damage is described as physical strand aberration caused by injury to the bond pattern, 8-hydroxydeoxyguanosine residues, and polycyclic aromatic hydrocarbon adducts as a result of DNA interactions with ROS or RNS. It is strongly recommended to eliminate free radicals from living systems using antioxidative enzymes (superoxide dismutase (SOD), catalase, and peroxidase) or chemical compounds such as ascorbic acid, tocopherol, and glutathione^5^. Antioxidant compounds have the intrinsic potential to counteract the negative effects of ROS and RNS. To reduce the risk of synthetic antioxidants, which are known to have negative effects on human health^6^, it has become more important to investigate alternative natural sources of antioxidants via food and beverages. Natural herbs, plants, and herbal products have been used for human health since ancient times, and the biological function of these herbal materials has been linked to the preservation of plant cells under stressful and abnormal conditions. Secondary metabolites, especially phenolic compounds and flavonoid compounds produced in plant cells, are known to protect plant function under adverse conditions^7,8^. Plants' antioxidant and antiradical effects are attributed to the presence of flavonoids, anthocyanins, and flavones^9^. Medicinal plants are thought to be a storehouse of bioactive substances, and they have taken centre stage as an ethnomedicinal use in humans, suggesting the most honoured one^10^. *Terminalia* plants are well-known for their antioxidant and biochemical properties in folklore all over the world^11^. For example, the  *T. chebula* plant has been stated to include healing, and it is known as the “King of Medicine” in Tibet^9,10^. *Terminalia arjuna* (arjuna) belongs to the Combretaceae family and is known as Arjuna, Dhavala, Kaubha, Nadisaraja, Veeravrikskha, Partha, and Indradru. It was recorded all over India, including the greater part of the Indian subcontinent, the Himalayan tract of Uttar Pradesh, Chota Nagpur, Orissa, West Bengal, Punjab, Deccan, and Konkan. *T. arjuna* bark has been shown to be effective as a cardioprotective and antihyperlipidemic medicine^11^. *T. arjuna*'s excellent phytochemical profiling and oxidative potential can draw attention to its use in humans for a variety of metabolic disorders^12,13^ and, to a lesser extent, in rodents^2,14–16^. While it has been extensively researched for its ethnomedicinal benefits, it has not been used in livestock or fish as a feed ingredient or in the formulation of medicated feed. Disease outbreaks and feed costs are the two major impediments to sustainable aquaculture development. The issues are divided into three categories: (1) long-term side effects of synthetic antioxidants, (2) short-term side effects of synthetic antioxidants, and (3) short-term side effects of synthetic antioxidants. Synthetic antioxidants are mostly used in the feed industry. Butylated hydroxy toluene (BHT), butylated hydroxy anilsole (BHA), and ethoxyquin are popular synthetic antioxidants used to preserve the freshness, flavour, and colour of foods and animal feeds, among other things. Synthetic antioxidants, such as BHT and BHA, have been shown to have tumor-promoting efficacy when used in high concentrations over long periods of time^17,18^. The discovery of new non-traditional plant-based feed ingredients provides a landscape for future studies in the area of feed and animal nutrition. (2) An increased risk of neurodegenerative chronic diseases caused by DNA damage. Malignancies caused by DNA damage are spreading rapidly around the world. The treatments for these cancers are either long-term steroids, chemotherapy, or organ transplants, which are risky at times and whose effectiveness is dependent on the person's age and immune status. A wealth of knowledge on *T. arjuna* solvent extracts of useful parts (bark, leaf, and fruit used in the current study) is dispersed and incomplete, which is critical for using the plant rationally in terms of phamacognosy. Plant-derived antioxidants have been shown to be an excellent source of natural antioxidants that are less expensive, acceptable, and consistent with animal physiology, and have fewer side effects than synthetic antioxidants. As a result, there is a quest to find and explore some novel herbal resources in order to grow natural livestock and fish for healthy humans. By referring to bibliographic studies on *T. arjuna's* ethnomedicinal, pharmacognostics, pharmacological, nutraceutical, and dietary results, it can be inferred that it has been explored for human beings, but has left a large scope for its exploration in live stocks and fish in terms of nutraceutical applications. Thus, research on these aspects may ascertain the effective valorization of solvent extracts of *T. arjuna* (arjuna) upon DNA scission and free radical scavenging to improve coupling responses and cognitive functions under in vitro conditions, which may pique the interest of researchers in further research on developing a remedy to eliminate or slow down the process of anarchy in humans. In this context, the current in vitro analysis was carried out to assess the phytochemical profiling, antioxidant, and DNA nicking inhibition capacity of selected *T. arjuna* solvent extracts.

## Results

### Qualitative screening of phytochemicals

The grouping of the solvent extracts was performed according to the phytochemical to be measured in order to derive phytochemicals. Based on their polarity, the solvents can be described as follows; Hexane < Ethyl acetate < Chloroform < Acetone < Ethanol < Methanol < Distilled water. The comparative qualitative phytochemical profile of *T. arjuna* solvent extracts was investigated using various phytochemical specific chemicals (Table 1). Color profiling of the solvent extract was also given for various tests (Supplementary file as Appendix A).

**Table 1:** Qualitative phytochemical analysis of solvent extracts of *T. arjuna*

Solvent/phytochemical	Tannin and phenolics	Steroids	Alkaloids	Flavonoids	Saponin
Hex-AB	−	−	−	−	−
Hex-AF	−	+	+++	−	−
Hex-AL	−	−	+	−	−
Etac-AB	−	+	++	+	−
Etac-AF	−	++	−	−	−
Etac-AL	+	−	−	−	−
Chlo-AB	−	−	−	−	−
Chlo-AF	−	+	+	−	−
Chlo-AL	+	−	+	−	+
Acet-AB	+++	+++	++	+	+
Acet-AF	++	++	+	+	+
Acet-AL	+++	+	ND	++	+++
Etoh-AB	+++	+++	++	+++	++
Etoh-AF	+++	+++	++	++	++
Etoh-AL	+++	++	+	++	++
Meoh-AB	+++	+++	+	++	++
Meoh-AF	+++	+++	++	++	+
Meoh-AL	+++	++	ND	++	+++
Dw-AB	++	+	−	+	+
Dw-AF	+	−	−	+	+
Dw-AL	++	ND	+	−	+

+++, high concentration; ++, moderate concentration; +, low concentration; ND, not detected, table showed that Etoh-AB showed a higher profile for tannins and phenolic, alkaloids and flavonoids. Similarly, high polar solvent extracts also showed better profiling as compared to apolar and aprotic solvent extracts.

### In vitro antioxidant activity

#### Estimation of FRAP

The FRAP activity for various solvent extracts was reported in the order as follows, bark > leaf > fruit. The capacity of bark extracts to reduce FRAP was found to be highest in ethanolic bark extract (2.5–34 M Fe(II)/g), followed by methanolic bark extract (Meoh-AB) (1.6–28.4 µM Fe(II)/g), acetone bark (Acet-AB) (2.4–20 µM Fe(II)/g), and distilled water extract of bark (Dw-AB) (1.5–14.5 µM Fe(II)/g).

The reducing capacity of the fruit extracts was greatest in methanolic fruit extract (0.98–9.9 µM Fe(II)/g) followed by acetone fruit extract (0.95–9.8 µM Fe(II)/g), Dw fruit extract (0.13–6.4 µM Fe(II)/g) and ethanolic fruit extract (0.10–2.2 µM Fe(II)/g). The reducing capacity of leaf extracts was highest in methanolic leaf extract (1.1–21.4 µM Fe(II)/g), followed by ethanolic leaf extract (Etoh-AL) (1.04–19.9 µm Fe(II)/g), acetone leaf extract (Acet-AL) (0.93–10.66 µM Fe(II)/g), and distilled water leaf extract (Dw-AL) (0.06–9.6 µM Fe(II)/g).

#### Estimation of total phenolic contents (TPC)

Total phenolic compound content differed according to solvent extract, with bark extract having the highest value, followed by fruit and leaf extract. Among bark extracts, ethanolic bark extract (144.67–1794 µg/mL gallic acid equivalent (GAE) was found to have the highest TPC value (144.67–1794 µg/mL GAE), followed by Meoh-AB (128–1211.33 µg/mL GAE), DW-AB (128–1228 µg/mL GAE), and Acet-AB (86–1003 µg/mL GAE). Among the fruit extracts, Etoh-AF had the highest TPC (103–1403 µg/mL GAE), followed by Dw-AF (111.33–1161.33 µg/mL GAE), Acet-AF (111.33–1078 µg/mL GAE), and Meoh-AF (111.33–1069.33 µg/mL GAE).

In the case of leaf extracts, Etoh-AL had the highest TPC (128–1303 g/mL GAE), followed by Meoh-AL (128–1186.33 g/mL GAE), Chlo-AL (11.33–873.89 g/mL GAE), and Dw-AL (78–786.33 g/mL GAE).

#### Total flavonoids content (TFC)

TFC of bark extracts was highest for Etoh-AB (52.7–382.7 µg/mL QE), followed by Meoh-AB (49.36–266.03 µg/mL QE), Dw-AB (49.36–229.37 µg/mL QE), and Acet-AB (41.03–224.37 µg/mL QE). Etoh-AF (44.37–304.37 µg/mL QE) was the most concentrated fruit extract, followed by Dw-AF (46.03–256.03 µg/mL QE), Acet-AF (46.03–239.37 µg/mL QE), and Meoh-AF (46.03–237.7 µg/mL QE). In leaf extracts, Etoh-AL had the highest TFC content (49.37–284.37 µg/mL QE), followed by Meoh-AL (49.37–261.03 µg/mL QE) and Chlo-AL (26.03–198.54 µg/mL QE).

#### DPPH inhibition activity

The DPPH inhibition (%) of bark showed maximum activity, Etoh-AB (36.44–73.78%) followed by Meoh-AB (24.66–72.44%), Acet-AB (14.44–65.11%) and Dw-AB (18.88–45.55%). For fruit extracts, maximum activity showed by Etoh-AF (22.35–72.84%) followed by Dw-AF (47.55–66.88%), Acet-AF (13.77–23.55%), and Meoh-AF (19.89–58.22%). In case of leaf extracts, Meoh-AL (24.46–71.18%) showed maximum activity followed by Etoh-AL (37.33–70%), Acet-AL (17.39–64.34%) and Dw-AL (16.27–35.93%).

#### 2,2′-Azino-bis-3-ethylbenzothiazoline-6-sulphonic acid (ABTS assay)

Comparative ABTS inhibition (%) potential of solvent extracts of *T. arjuna* is depicted in Fig. 5. Etoh-AB showed maximum ABTS inhibition (34.31–71.64%) followed by Meoh-AB (22.64–70.53%), Acet-AB (12.31–62.98%) and Dw-AB (16.75–38.42%). For fruit extracts, maximum activity showed by Etoh-AF (20.8–71.24%) followed by Dw-AF (46.35–65.68%), Acet-AF (12.57–62.35%), and Meoh-AF (18.35–57.02%). In case of leaf extracts, Etoh-AL (32.85–67.52%) followed by Meoh-AL (21.74–65.40%) showed maximum activity, Acet-AL (14.63–41.52%) and Dw-AL (12.52–33.29%).

#### Nitric oxide assay (NO assay)

For NO inhibition assay, Etoh-AB showed maximum value (39.64–76.97%) followed by Meoh-AB (26.06–75.36%), Acet-AB (17.64–68.51%) and Dw-AB (22.28–43.95%). For fruit extracts, maximum activity showed by Etoh-AF (24.89–75.35%) followed by Dw-AF (50.44–69.77%), Acet-AF (16.67–66.44%), and Meoh-AF (22.35–61.02%). In case of leaf extracts, Etoh-AL showed maximum activity (38.53–72.25%) followed by Meoh-AL (27.74–70.43%) Acet-AL (20.63–45.52%) and Dw-AL (17.52–38.29%).

#### DNA nicking inhibition activity

**Figure 1:**
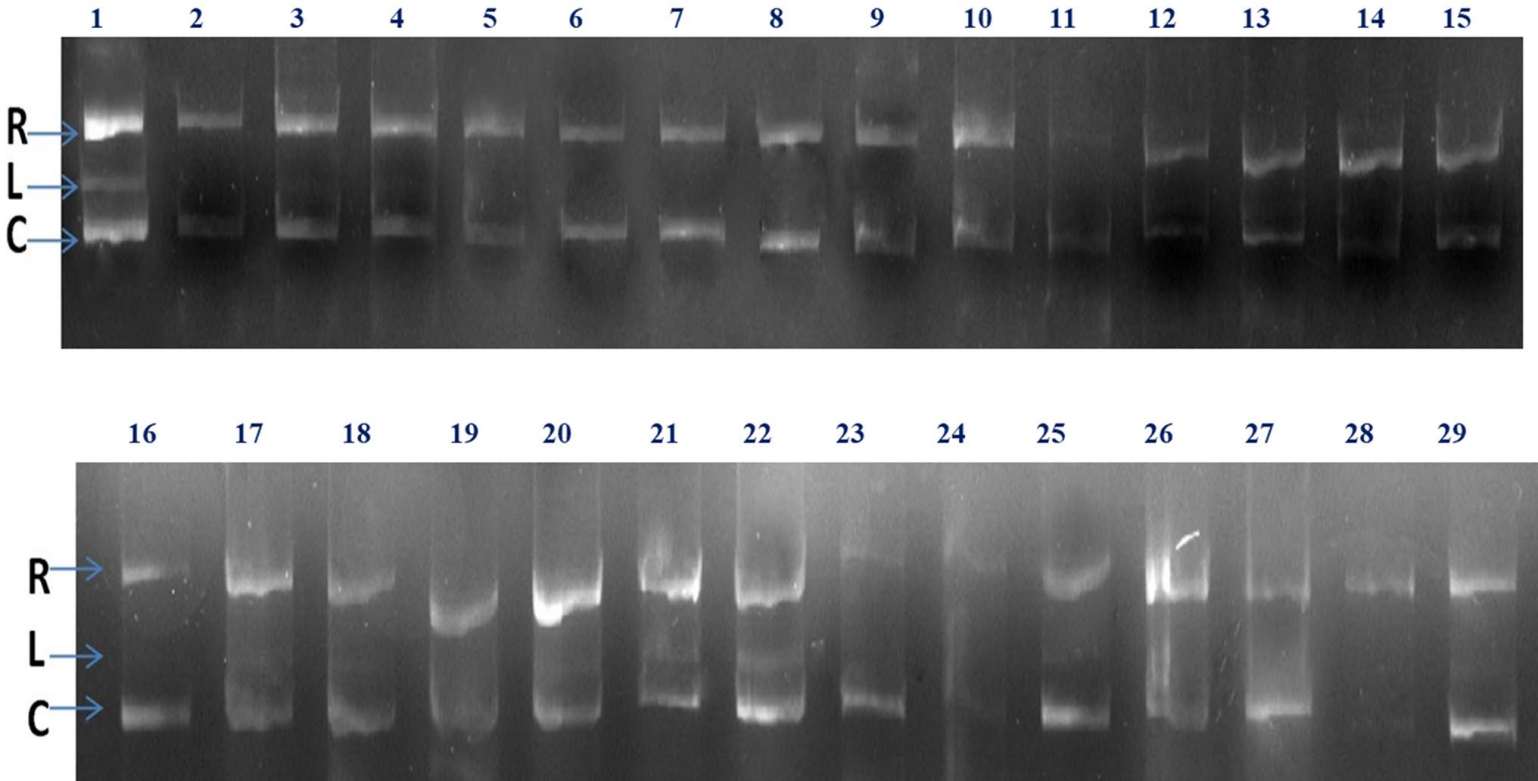
Showing comparative electrophoretic pattern of pBR322 DNA nicking inhibition activity of solvent extracts of *T. arjuna.* Here, 1–29 is representating lanes: 1—Plasmid DNA; 2—H_2_O_2_ + Plasmid DNA; 3—THF + Plasmid DNA; 4—THF + H_2_O_2_ + Plasmid DNA; 5—BHA + H_2_O_2_ + Plasmid DNA; 6—BHT + H_2_O_2_ + Plasmid DNA; 7—Ascorbic acid + H_2_O_2_ + Plasmid DNA; 8—Gallic acid + H_2_O_2_ + Plasmid DNA; 9—Hex-AB H_2_O_2_ + Plasmid DNA; 10—Etac-AB H_2_O_2_ + Plasmid DNA; 11—Chlo-AB H_2_O_2_ + Plasmid DNA; 12—Acet-AB H_2_O_2_ + Plasmid DNA; 13—Etoh-AB H_2_O_2_ + Plasmid DNA; 14—Meoh-AB H_2_O_2_ + Plasmid DNA; 15—Dw-AB H_2_O_2_ + Plasmid DNA;16—Hex-AL H_2_O_2_ + Plasmid DNA; 17—Etac-AL H_2_O_2_ + Plasmid DNA; 18—Chlo-AB H_2_O_2_ + Plasmid DNA; 19—Acet-AL H_2_O_2_ + PlasmidDNA; 20—Etoh-AL + H_2_O_2_ + Plasmid DNA; 21—Meoh-AL + H_2_O_2_ + Plasmid DNA; 22—Dw-AL + H_2_O_2_ + Plasmid DNA; 23—Hex-AF + H_2_O_2_ + Plasmid DNA; 24—Etac-AF + H_2_O_2_ + Plasmid DNA; 25—Chlo-AF + H_2_O_2_ + Plasmid DNA; 26—Acet-AF + H_2_O_2_ + Plasmid DNA; 27—Etoh-AF + H_2_O_2_ + Plasmid DNA; 28—Meoh-AF + H_2_O_2_ + Plasmid DNA; 29—Dw-AF + H_2_O_2_ + Plasmid DNA. Lanes 9–29 containing sample. The control sample in lane 1 contains plasmid DNA and thus forming the reference band as R-relaxed or super coiled form which migrates faster in gel, L-linear, C-coiled forms of plasmid DNA highlighted plasmid DNA interacted with a fixed concentrations of the selected solvent extracts of *T. arjuna* extracts in H_2_O_2_ condition.

Figure 1 depicts the comparative DNA nicking inhibition behaviour of solvent extracts. Lanes 1–4 are sensors, and lanes 5–8 are standard antioxidants used to measure and compare the effectiveness of extracts. The control sample in lane 1 plasmid DNA, thus forming the reference band as R-relaxed or super coiled type of plasmid DNA which migrates faster in gel, L-linear, C-coiled forms of plasmid DNA, while lanes 9–29 containing sample highlighted plasmid DNA interacted with a fixed concentrations of the selected solvent extracts of *T. arjuna* in H2O2 condition. Effective solvent extracts had a protective effect on hydroxyl radical-mediated plasmid DNA damage, whereas ineffective solvent extracts had no protective effect on plasmid DNA in H2O2 conditions, as evidenced by the ruptured or smeared gel image of the respective extracts, which occurred in the case of non-polar and, to a lesser extent, polar aprotic solvent extracts. Gallic acid had the highest DNA nicking inhibition activity of the four standards tested, followed by ascorbic acid > BHT > BHA. According to the gel, Etoh-AB, Meoh-AB, and Acet-AB have the highest activity, followed by Etoh-AF, Acet-AF, Chlo-AL, Etac-AL, and others, all of which have relatively similar effects on plasmid DNA. Quantitatively, the protective effects can be seen in the relative front value (RF), relative quantity (RQ), band (%), and lane (%) values. The RF values vary insignificantly (*p* > 0.05) when compared to the standard controls, with the highest value reported for Etoh-AB, followed by (0.66 ± 0.13), Meoh-AB (0.66 ± 0.11), Acet-AB (0.62 ± 0.14), and Dw-AB (0.61 ± 0.14), respectively. A similar pattern was observed for fruit and leaf solvent extracts. The pattern for RQ value was the same, with maximum values for Etoh-AB (1.20 ± 0.29 ng), Meoh-AB (1.16 ± 0.30 ng), Acet-AB (0.97 ± 0.34 ng) and Dw-AB (0.84 ± 0.37 ng), respectively, and almost the same trend was observed for fruit except for the Dw and Acetone fractions, but for leaf the trend changes depending on operation. For RQ, there was a substantial difference (*p* < 0.05) between Etoh-AB and Dw-AB. One-way ANOVA revealed 11 and 12 solvent extract groups for band (%) and lane (%), respectively, indicating major differences between solvent extracts (Table 2). The band (%) was found to be largest in Etoh-AB (46 ± 0.03), followed by Meoh-AB (44.9 ± 0.03), Acet-AB (39.7 ± 0.03) and Dw-AB (36.8 ± 0.03), in that order. Lane (%) varied substantially (*p* < 0.05) in ascending order, as follows: 20.90.05 >, 19.20.06 >, 16.80.06 >, and 15.60.04 for Etoh-AB, Meoh-AB, Acet-AB, and Dw-AB.

**Table 2:** Densiometric analysis of gel image of DNA nicking inhibition by solvent extracts of *T .arjuna*

Gel Coding	Abbreviation	Rf	RQ (ng)	Band (%)	Lane (%)
L1	Plasmid DNA	0.87 ± 0.13^d^	1.32 ± 0.26^c^	56.8 ± 0.02^i^	28.3 ± 0.04^h^
L2	H_2_O_2_ + Plasmid DNA	0.55 ± 0.17^a^	0.33 ± 1.05^a^	3.9 ± 0.32^a^	4.2 ± 0.22^a^
L3	THF + Plasmid DNA	0.61 ± 0.15^a^	0.8 ± 0.39^ab^	35.7 ± 0.03^bcd^	14.4 ± 0.06^de^
L4	H_2_O_2_ + THF + + Plasmid DNA	0.57 ± 0.17^a^	0.35 ± 1.05^a^	4.1 ± 0.32^a^	4.4 ± 0.22^a^
L5	BHA + H_2_O_2_ + Plasmid DNA	0.63 ± 0.15^a,c^	0.83 ± 0.39^ab^	37.7 ± 0.03^a^	16.4 ± 0.06^de^
L6	BHT + H_2_O_2_ + Plasmid DNA	0.66 ± 0.15^c^	0.89 ± 0.39^ab^	38.5 ± 0.03^abc^	17.2 ± 0.06^de^
L7	Ascorbic acid + H_2_O_2_ + Plasmid	0.73 ± 0.15^b^	0.95 ± 0.39^ab^	52.6 ± 0.03^bci^	22.2 ± 0.06^de^
L8	Gallic acid + H_2_O_2_ + Plasmid DNA	0.84 ± 0.15^a^	1.26 ± 0.39^ab^	54.7 ± 0.03^a,d,i^	24.2 ± 0.06^de^
L9	H_2_O_2_ + Plasmid DNA + Hex-AB	0.56 ± 0.16^c,d^	0.32 ± 1.05^a^	4.9 ± 0.25^a^	3.3 ± 0.28^a^
L10	H_2_O_2_ + Plasmid DNA + Etac-AB	0.59 ± 0.14^c,d^	0.83 ± 0.39^b^	37.3 ± 0.03^cde^	13.2 ± 0.07^c^
L11	H_2_O_2_ + Plasmid DNA + Chlo-AB	0.32 ± 0.14^b^	0.2 ± 0.39^a,c^	21.8 ± 0.03^bc^	3.2 ± 0.10^b^
L12	H_2_O_2_ + Plasmid DNA + Acet-AB	0.62 ± 0.14^c^	0.97 ± 0.34^b^	39.7 ± 0.03^ij^	16.8 ± 0.06^de^
L13	H_2_O_2_ + Plasmid DNA + Etoh-AB	0.68 ± 0.13^a^	1.23 ± 0.29^b^	49 ± 0.03^j^	22.9 ± 0.05^gh^
L14	H_2_O_2_ + Plasmid DNA + Meoh-AB	0.66 ± 0.11^a^	1.16 ± 0.30^b^	44.9 ± 0.03^ij^	19.2 ± 0.06^fg^
L15	H_2_O_2_ + Plasmid DNA + Dw-AB	0.61 ± 0.14^a^	0.84 ± 0.37^a^	36.8 ± 0.03^hi^	15.6 ± 0.04^jkl^
L16	H_2_O_2_ + Plasmid DNA + Hex-AL	0.63 ± 0.13^a^	1.01 ± 0.27^b^	45.1 ± 0.03^j^	20.8 ± 0.04^jkl^
L17	H_2_O_2_ + Plasmid DNA + Etac-AL	0.63 ± 0.14^a^	0.86 ± 0.37^ab^	34 ± 0.04^b^	13.6 ± 0.07^cd^
L18	H_2_O_2_ + Plasmid DNA + Chlo-AL	0.61 ± 0.15^a^	1.09 ± 0.29^b^	42 ± 0.03^h^	19.9 ± 0.05^ijkl^
L19	H_2_O_2_ + Plasmid DNA + Acet-AL	0.59 ± 0.15^a^	0.95 ± 0.33^b^	38.7 ± 0.03f.	14.5 ± 0.06^de^
L20	H_2_O_2_ + Plasmid DNA + Etoh-AL	0.65 ± 0.14^a^	1.03 ± 0.31^b^	41 ± 0.03^gh^	20 ± 0.05^ijkl^
L21	H_2_O_2_ + Plasmid DNA + Meoh-AL	0.54 ± 0.14^a^	0.97 ± 0.32^b^	37.3 ± 0.01^def^	13.9 ± 0.04^d^
L22	H_2_O_2_ + Plasmid DNA + Dw-AL	0.61 ± 0.15^a^	0.82 ± 0.38^ab^	39.6 ± 0.03^fg^	18.5 ± 0.05^hi^
L23	H_2_O_2_ + Plasmid DNA + Hex-AF	0.61 ± 0.15^a^	0.92 ± 0.34^b^	35.3 ± 0.03^bc^	15.6 ± 0.06^ef^
L24	H_2_O_2_ + Plasmid DNA + Etac-AF	0.60 ± 0.15^a^	0.81 ± 0.39^ab^	38.3 ± 0.03^ef^	19.3 ± 0.05^ij^
L25	H_2_O_2_ + Plasmid DNA + Chlo-AF	0.60 ± 0.14^a^	0.89 ± 0.35^ab^	37.9 ± 0.03^bc^	13.3 ± 0.07^cd^
L26	H_2_O_2_ + Plasmid DNA + Acet-AF	0.61 ± 0.14^a^	0.95 ± 0.37^ab^	38.8 ± 0.03^def^	19.3 ± 0.05^fg^
L27	H_2_O_2_ + Plasmid DNA + Etoh-AF	0.64 ± 0.14^a^	1.01 ± 0.31^b^	45.4 ± 0.03^j^	20.8 ± 0.04^jkl^
L28	H_2_O_2_ + Plasmid DNA + Meoh-AF	0.59 ± 0.14^a^	0.85 ± 0.33^b^	37.6 ± 0.03f.	14.7 ± 0.04^jkl^
L29	H_2_O_2_ + Plasmid DNA + Dw-AF	0.62 ± 0.15^a^	0.99 ± 0.32^b^	39.4 ± 0.03^fg^	19.8 ± 0.05^ijk^

Different superscript in same column indicate significant difference at *p* < 0.05. Table showing, the result of densitometry analyses in H_2_O_2_ conditions. Table showed numerical values of the standard antioxidants for densitometry parameters in the order of Gallic acid > Ascorbic acid > BHT and BHA, while, control has more values. Among, solvent extracts Etoh-AB has significantly (*p* < 0.05) highest values as compared to non-polar and polar aprotic solvents extracts. There is no significant (*p* > 0.05) difference was observed between Etoh-AB, Meoh-AB and Etoh-AF, Meoh-Af and DW-AF.

### Over all association patterns, significance, networking and ordination scaleogram among solvent extracts based on antioxidant potential

**Figure 2:**
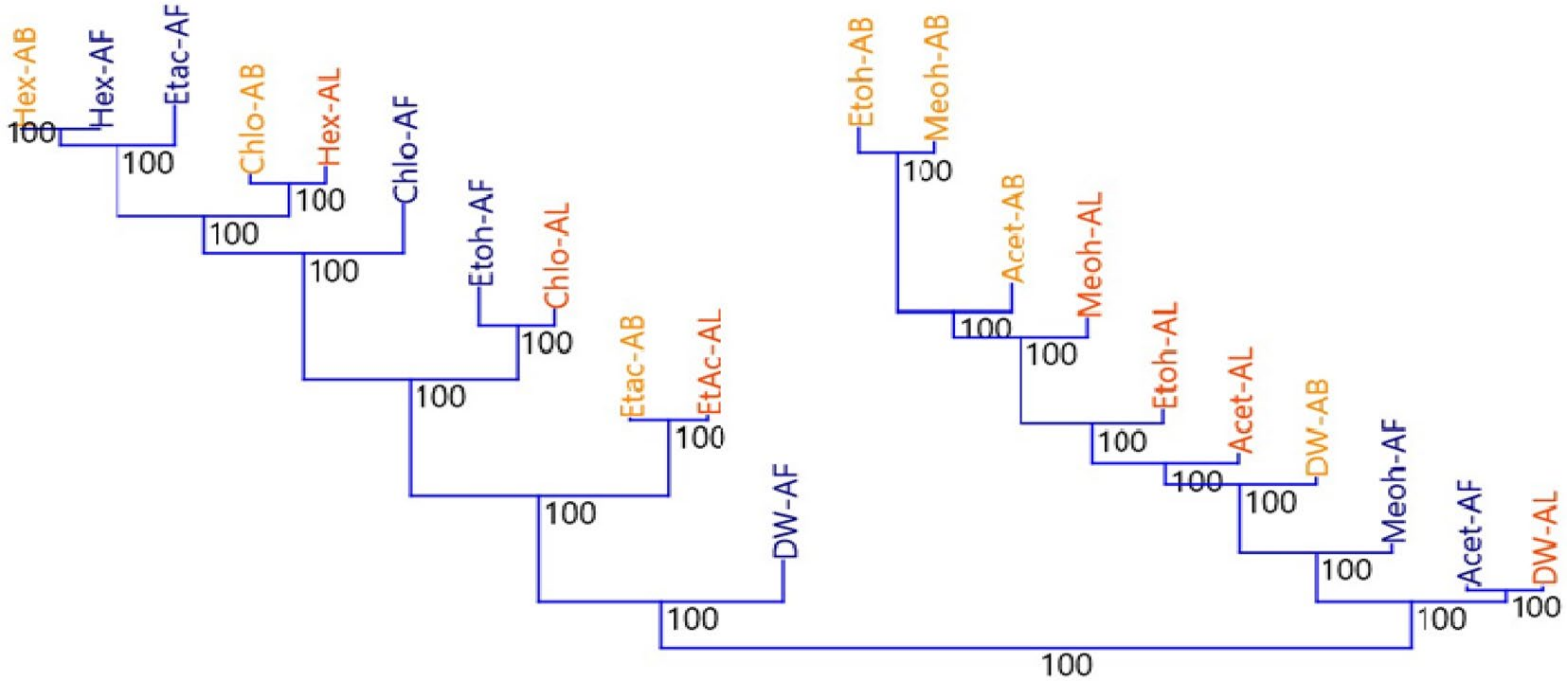
Association pattern between solvent extracts of *T. arjuna* based on the antioxidant potential. Two variables are included in the analysis: the bootstrap value and the distribution similarity index. Figure showing two main cluster and thereafter each custer divided into subcluters based on their antioxidant potential. Cluster one includes mainly polar and polar aprotic solvent extracts except DW-AF and Etoh-AF. Categorically, Etoh-AB and Meoh-AB showed same pattern, followed by Acet-AB > Meoh-AL > Etoh-AL > Acet-AL > DW-AB > Meoh-AF > Acet-AF > DW-AL. In second cluster Hex-AB, Hex-Af and Etac-AF showed same pattern of distribution.

The antioxidant ability of the solvent extracts is used to network. Dw-AL in a centric role, separating extracts of high polar and non-polar or polar aprotic solvents. Solvent extracts with high antioxidant potential are distributed on the far left hand side, while less active solvent extracts are distributed on the far right hand side (Fig. 2).

**Figure 3:**
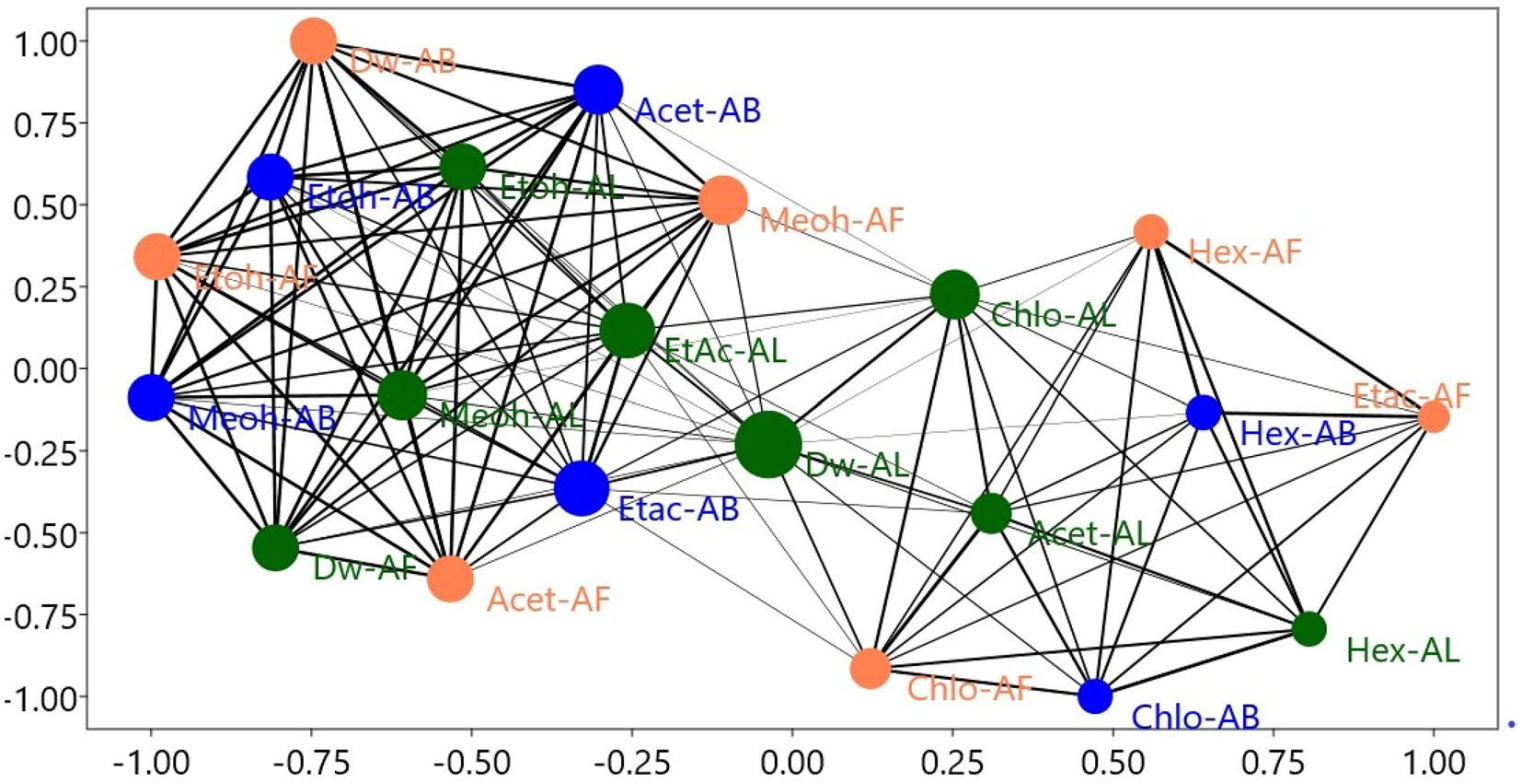
Showing interaction networking of solvent extract of *T. arjuna.*

Solvent extract networking at 50% edge cutoffs reveals more than 50% similarity between connecting edges of nodes. Node diameter is proportional to the number of edges connected to it, and edge thickness is proportional to similarity. Starting from the extreme right, the antioxidant activities of Hex-AL, Etac-AF, Chlo-AB, Chlo-AL, Chlo-AF, Acet-AL, and Hex-AL are more than 50% identical. On the other hand, Dw-AL has 19 connecting edges that bind the aforementioned group to high polar solvent extracts such as Etac-AB, Acet-AF, Etac-AL, Acet-AB, Etoh-AL, Meoh-AL, Dw-AF, Dw-AB, Etoh-AB, Meoh-AB. The linking edges of Chlo-AB and Hex-AF are 7, 7, 7, 8 and 8, respectively. Other solvent extracts with common similarity patterns include Acet-AF, Dw-AF, Meoh-AB, Etoh-AB, and Etoh-AF (Fig. 3).

The correlation degree that DPPH has a highly important (P0.01) positive correlation with other antioxidants is as follows in ascending order: ABTS > NO scav. > TPC > FLV > FRAP, which can be expressed in (%) correlation as 99.1, 99 > 83 > 77.4 > 69.3, respectively. Similarly, ABTS and NO scav. (%) showed a correlation in the same way that DPPH did. FRAP, on the other hand, displayed a highly important (*p* = 0.01) positive correlation with all antioxidants, with the highest correlation with DPPH (0.693). TPC and FLV displayed a highly significant (*p* < 0.01) positive association with each other, in comparison to FRAP and scavenging antioxidants such as DPPH, ABTS, and NO (Table 3).

**Table 3:** Correlation matrix between antioxidants based on the solvent extracts reactions at *p* < 0.01 level

	DPPH	FRAP	ABTS	NO SCAV	FLV	TPC
DPPH	1	0.693**	0.991**	0.990**	0.774**	0.830**
*p* value		0.001	0.001	0.001	0.001	0.001
FRAP	0.693**	1	0.681**	0.689**	0.591**	0.630**
*p* value	0.001		0.001	0.001	0.001	0.001
ABTS	0.991**	0.681**	1	1.000**	0.787**	0.844**
*p* value	0.001	0.001		0.001	0.001	0.001
NOSCAV	0.990**	0.689**	1.000**	1	0.787**	0.845**
*p* value	0.001	0.001	0.001		0.001	0.001
FLV	0.774**	0.591**	0.787**	0.787**	1	0.974**
*p* value	0.001	0.001	0.001	0.001		0.001
TPC	0.830**	0.630**	0.844**	0.845**	0.974**	1
*p* value	0.001	0.001	0.001	0.001	0.001	

**Correlation is significant at the 0.01 level.

The correlation level that DPPH has highly significant (*p* < 0.01) positive correlation with other antioxidant which is as follows in ascending order ABTS > NO scav. > TPC > FLV > FRAP which can be apparently expressed in (%) correlation as 99.1 > 99 > 83 > 77.4 > 69.3, respectively. Similarly, ABTS and NO scav. (%) also showed the correlation in same fashion as in case of DPPH. Whereas FRAP showed highly significant (*p* < 0.01) positive correlation with all antioxidants and the showed the maximum correlation with DPPH (0.693). In contrast to FRAP and Scavenging antioxidants such as DPPH, ABTS and NO, TPC and FLV showed highly significant (*p* < 0.01) positive correlation with each other (Table 3).

Etoh-AB has the highest numerical value of 23.7, followed by Meoh-AB and Acet-AB. Dw counter parts had the lowest values. According to the PCA scatter bi-plot, PCA components 1 and 2 explained 84.54 and 12.52% of the difference, respectively (Fig. 5). The antioxidant potential of solvent extracts Etoh-AB >, Meoh-AB > Acet-AB > Meoh-AL is strongly associated with DPPH, ABTS, NO scavenging activity, and FRAP potential in ascending order. TPC and FLV, on the other hand, are highly intracorrelated with the operation of Dw-AF >, Etoh-AL > Meoh-AF > Etoh-AF > Acet-AF. On the other side of the axis, solvent extracts such as Chlo-AL and Chlo-AF are strongly correlated with each other but have no correlation with regular antioxidants. Similarly, the association between Dw-AL, Acet-AL, Hex-AL, Chlo-AB, Hex-AB, Hex-AF, and Etac-AF and standard antioxidants is weak.

**Figure 4:**
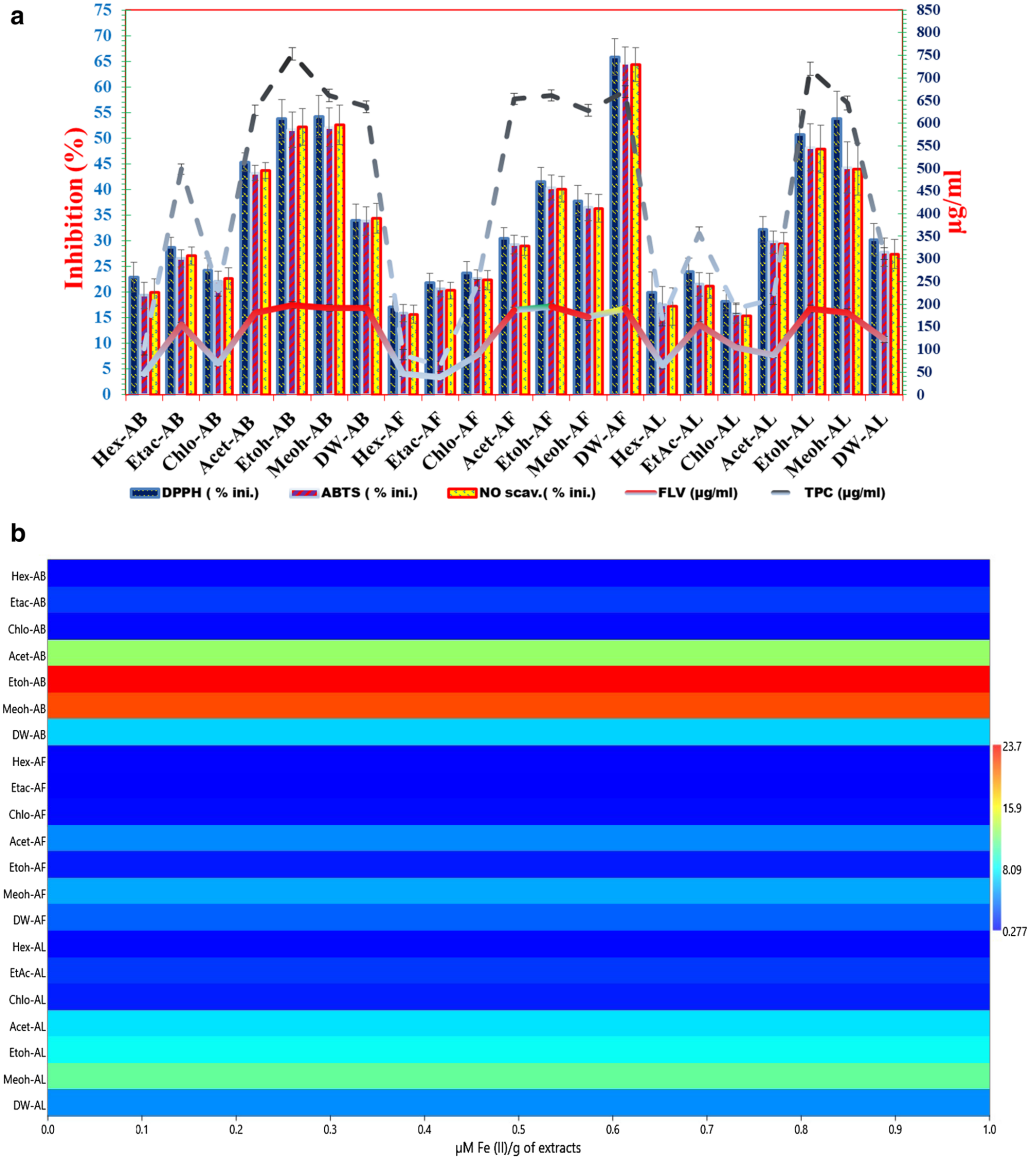
(**a**) Combined effects of solvent extracts based on antioxidants potential. The values of DPPH, ABTS and NO scavenging are represented in % where as FLV and TPC being represented in µg/mL. The comparative multiple bar of solvent extracts showed that Etoh-AB had the highest antioxidant and scavenging activity as compared to the solvent extracts. Dw-AF is the most effective among fruit extracts, and Etoh-AL is the most effective among leaf extracts (**a**). The comparative FRAP capacity of the extracts ranged from 0.277 to 23.7 µM Fe(II)/g of extracts (**b**). (**b**) Comparative effects of solvent extracts based on FRAP potential.

**Figure 5:**
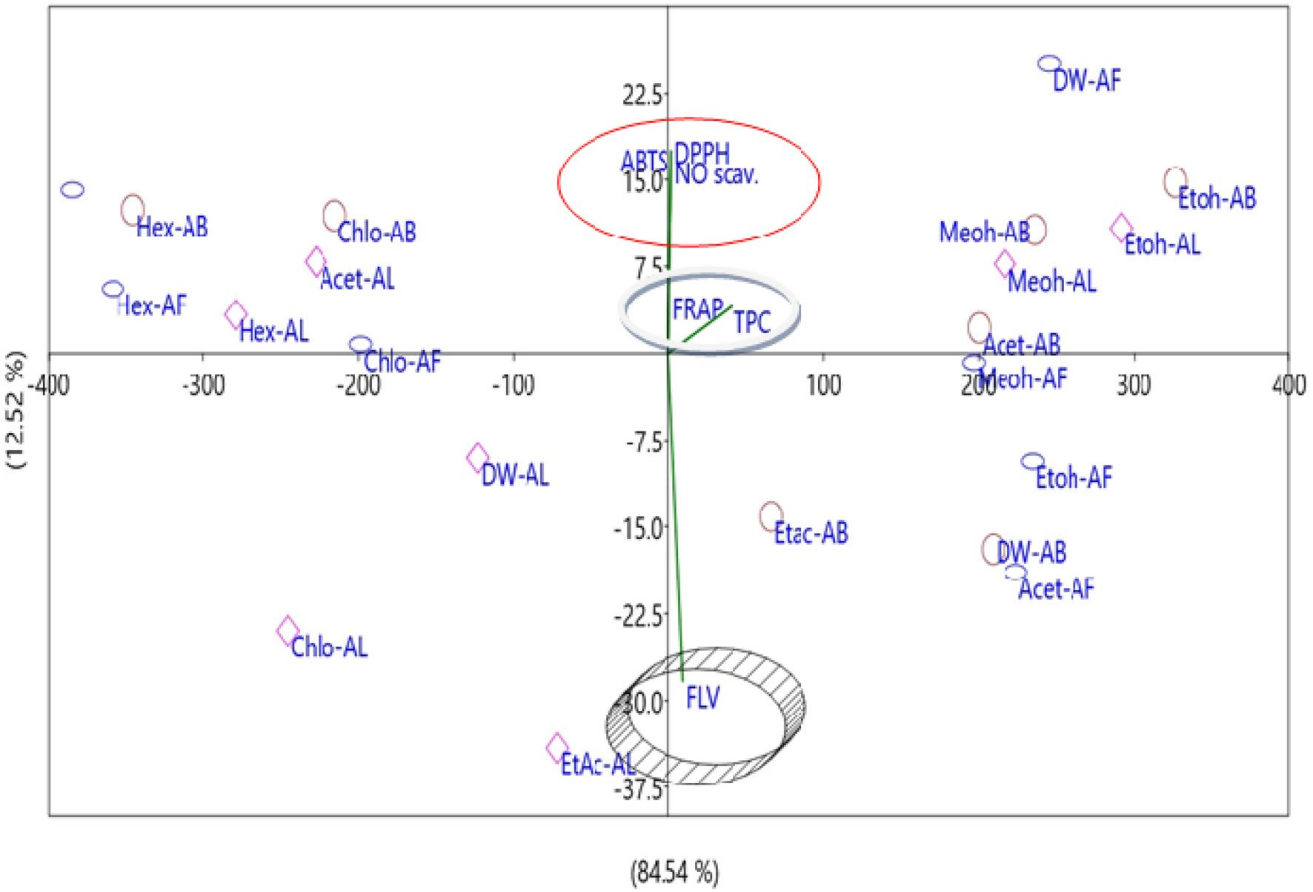
PCA scatter bi-plot of Antioxidants and solvent extracts of *T. arjuna*. Components 1 and 2 describe differences depending on the Eigen values of the various components. Component 1 describes the overall variance, which shows the best fit of the experimental results. FRAP is closely related to Etoh-AB and Meoh-AB, while TPC is related to Etoh-AF and Meoh-AF. TPC is closely linked to FLV material, while FRAP is strongly associated with DPPH, ABTS, and NO.

### Phytochemical analysis using Liquid chromatography coupled to electrospray-Orbitrapmass spectrometry

**Figure 6:**
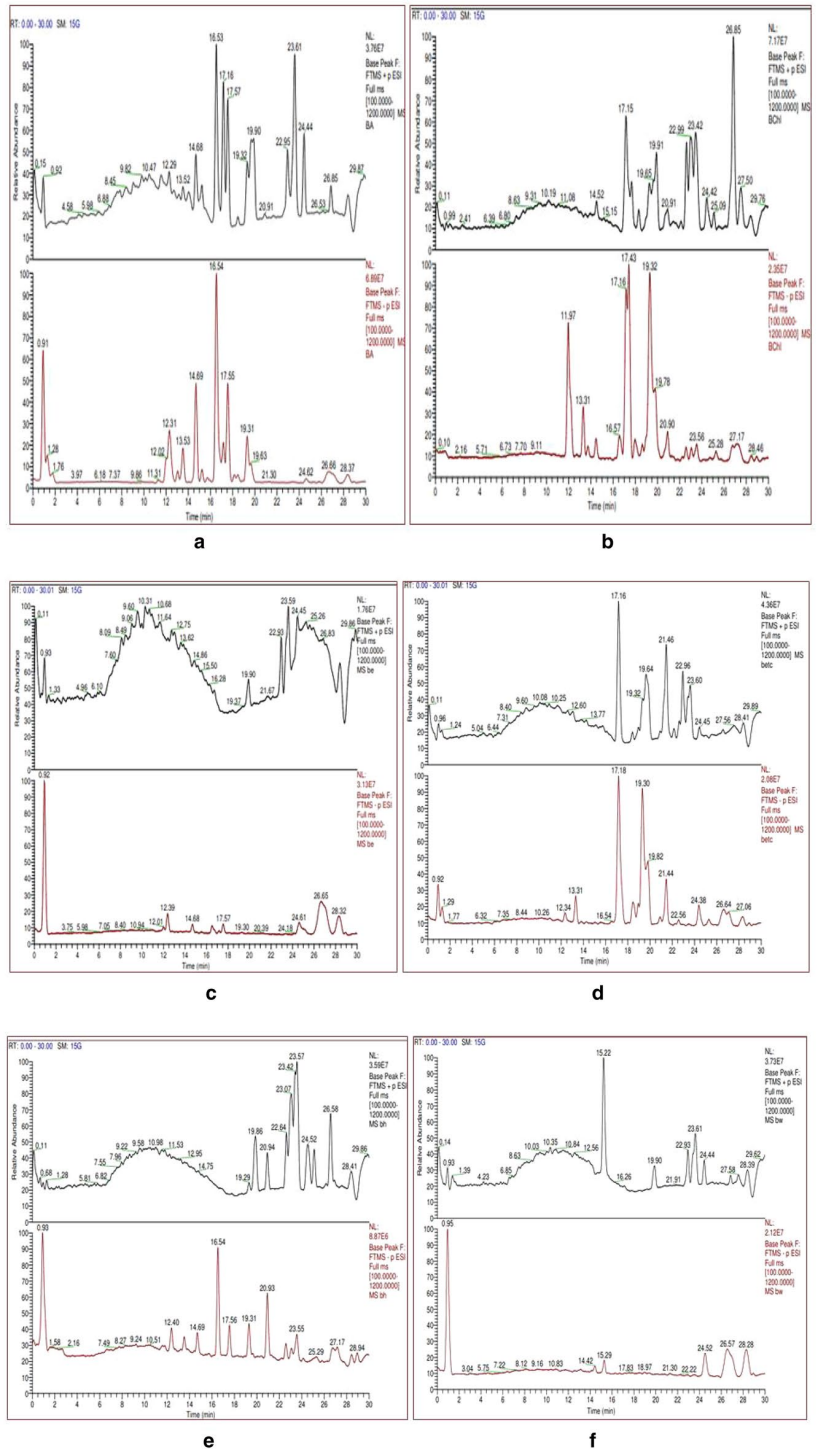

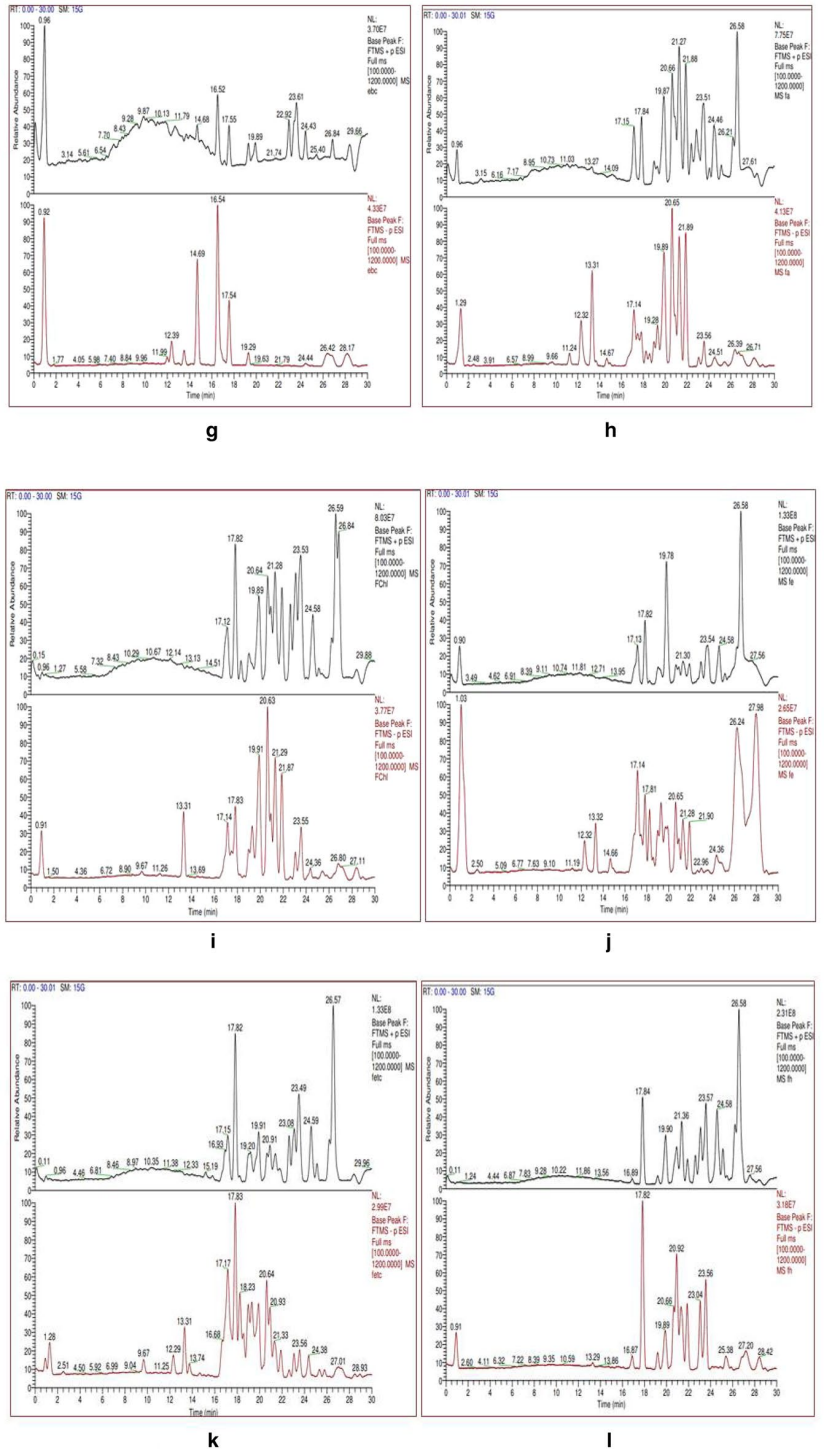

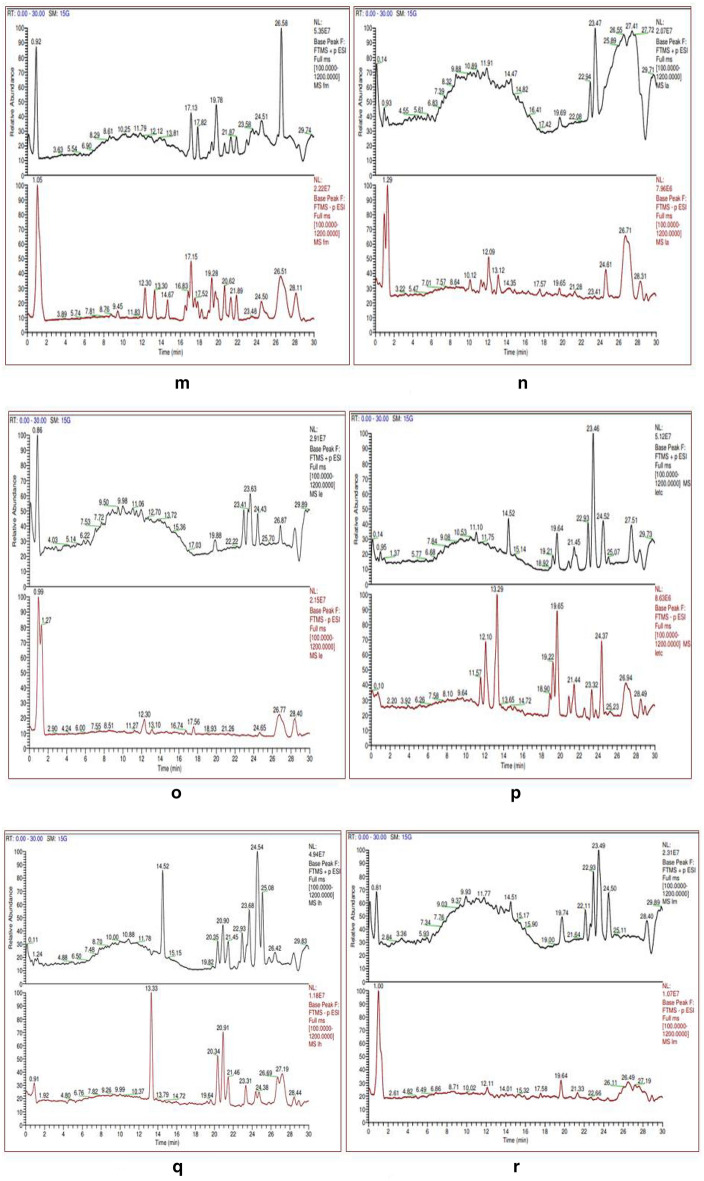
(**a**) Chromatogram for Acet-AB and (**b**) for Chlo-AB. (**c**) Chromatogram for Etoh-AB and (**d**) for Dw-AF. (**e**) Chromatogram for Hex-AB and (**f**) for Meoh-AB. (**g**) Chromatogram for Dw-AB and (**h**) for Acet-AF. (**i**) Chromatogram for Chlo-AF and (**j**) for Etoh-AF. (**k**) Chromatogram for Etac-AF and (**l**) for Hex-AF. (**m**) Chromatogram for Meoh-AF and (**n**) for Acet-AL. (**o**) Chromatogram for Etoh-AL and (**p**) for Etac-AL. (**q**) Chromatogram for Hex-AL and (**r**) for Meoh-AL.

The data obtained from anQ-Exactive plus-Orbitrap hybrid mass spectrometer showed elution profile of different metabolites as their retention time Fig. 6a–r.

For thorough study, the samples were divided into groups, such as bark, leaf, and fruit, and individual samples were examined within each group. Gallic acid (*m/z* 170.0208 g/mol) and Ellagic acid (*m/z* 302.0063 g/mol) were found in three fruit extracts (FA, FE, FETC) out of a total of six, three leaf extracts (LA, LE, LM) out of a total of five, and five bark extracts (BA, BE, BETC, BM, EBC) out of a total of eight. Gallic acid, also known as 3,4,5-trihydroxybenzoic acid, is a hydrolysable tannin. Ellagic acid is a dimer of gallic acid formed by oxidative aromatic coupling dimerization. It functions as an antioxidant and can be found in a variety of fruits and leaf extracts. Ursolic acid, a triterpene metabolite, was also present in high concentrations in bark extracts. Ursolic acid has a molecular weight of 456.3609 g/mol and is also known as 3 β,-hydroxy-urs-12-en-28-oic acid. It is a triterpenoid with a C-30 chemical structure made up of isoprenoid units and a pentacyclic loop. Myricetin and quercetine were discovered in some of the bark and leaf extracts as flavonoid compounds. Table 4 summarises the metabolites found in bark, leaf, and fruit extracts.

**Table 4:** Bioactive compounds identified/screened from different solvent extracts of *Terminalia Arjuna*

Sample code	Compound name	Molecular formula	Molecular weight	Area
Acet-AB	Gallic Acid	C7 H6 O5	170.0208	3,751,314
	Ellagic acid	C14 H6 O8	302.0064	6,240,864
	(-)-Epigallocatechin	C15 H14 O7	306.0736	4,666,503
	Catechin	C15 H14 O6	290.0779	830,681
	Catechin gallate	C22 H18 O10	442.0895	534,767
	Quercetin	C15 H10 O7	302.0422	298,464
	Epicatechin	C15 H14 O6	290.0794	1,191,073
	Epigallocatechin gallate	C22 H18 O11	458.0844	785,517
	Myricetin	C15 H10 O8	318.0370	424,585
	Ursolic acid	C30 H48 O3	456.3609	366,622
Etac-AB	18-β-Glycyrrhetinic acid	C30 H46 O4	470.3400	6,086,264
	Ellagic acid	C14 H6 O8	302.0066	800,504
	Gallic acid	C7 H6 O5	170.0208	1,413,705
	Jasmonic acid	C12 H18 O3	210.1259	127,262
	Quercetin-3β-D-glucoside	C21 H20 O12	464.0938	173,239
	Ursolic acid	C30 H48 O3	456.3583	343,487
Meoh-AB	18-β-Glycyrrhetinic acid	C30 H46 O4	470.3404	1,130,958
	Ellagic acid	C14 H6 O8	302.0067	3,237,724
	Gallic acid	C7 H6 O5	170.0209	858,510
	Jasmonic acid	C12 H18 O3	210.1260	100,145
	Quercetin-3β-D-glucoside	C21 H20 O12	464.0962	59,251
Etoh-AB	Catechin	C15 H14 O6	290.0794	485,673
	Ellagic acid	C14 H6 O8	302.0064	1,417,504
	Gallic acid	C7 H6 O5	170.0208	555,546
	(–)-Epigallocatechin	C15 H14 O7	306.0734	2,348,791
	18-β-Glycyrrhetinic acid	C30 H46 O4	470.3400	6,672,616
Chlo-AB	Betulin	C30 H50 O2	442.3821	535,267
	Jasmonic acid	C12 H18 O3	210.1260	440,310
	Ursolic acid	C30 H48 O3	456.3585	1,065,991
Hex-AB	Cholest-4-en-3-one	C27 H44 O	384.3398	237,038
	Gallic acid	C7 H6 O5	170.0209	190,613
	Jasmonic acid	C12 H18 O3	210.1259	154,867
	Tropine	C8 H15 N O	141.1158	97,383
	Betulin	C30 H50 O2	442.3815	47,454
	Ursolic acid	C30 H48 O3	456.3608	977,223
Dw-AB	Hesperetin	C16 H14 O6	302.0792	1,673,957
	4-Hydroxycoumarin	C9 H6 O3	162.0319	386,621
	4-Methoxycinnamic acid	C10 H10 O3	160.0527	150,602
	Quinine	C20 H24 N2 O2	324.1839	661,869
Etac-AL	Quercetin-3β-D-glucoside	C21 H20 O12	464.0965	525,241
	Orientin	C21 H20 O11	448.1012	245,718
	Tropine	C8 H15 N O	141.1157	103,342
	Jasmonic acid	C12 H18 O3	210.1259	978,653
	Gallic acid	C7 H6 O5	170.0208	417,073
	Andrographolide	C20 H30 O5	350.2076	303,040
	18-β-Glycyrrhetinic acid	C30 H46 O4	470.3404	875,408
Hex-AL	Vitexin	C21 H20 O10	432.1062	2,593,852
	Ursolic acid	C30 H48 O3	456.3588	90,132
	Jasmonic acid	C12 H18 O3	210.1261	979,318
	Andrographolide	C20 H30 O5	350.2077	463,489
	18-β-Glycyrrhetinic acid	C30 H46 O4	470.3407	179,3617
Acet-AL	Quercitrin	C21 H20 O11	448.1013	823,611
	Gallic acid	C7 H6 O5	170.0208	2,825,765
	Ellagic acid	C14 H6 O8	302.0065	573,841
	Orientin	C21 H20 O11	448.1012	219,466
	Myricetin	C15 H10 O8	318.0377	206,708
	Jasmonic acid	C12 H18 O3	210.1260	341,528
	18-β-Glycyrrhetinic acid	C30 H46 O4	470.3404	240,403
Etoh-AL	Quercetin-3β-D-glucoside	C21 H20 O12	464.0953	793,870
	Orientin	C21 H20 O11	448.1014	132,684
	Jasmonic acid	C12 H18 O3	210.1260	387,114
	Myricetin	C15 H10 O8	318.0380	163,504
	Gallic acid	C7 H6 O5	170.0208	6,062,407
	Epigallocatechin gallate	C22 H18 O11	458.0845	755,144
	Ellagic acid	C14 H6 O8	302.0067	1,045,378
	18-β-Glycyrrhetinic acid	C30 H46 O4	470.3408	140,490
Meoh-AL	18-β-Glycyrrhetinic acid	C30 H46 O4	470.3405	90,373
	Ellagic acid	C14 H6 O8	302.0066	452,955
	Gallic acid	C7 H6 O5	170.0208	1,994,162
	Jasmonic acid	C12 H18 O3	210.1258	251,721
	Orientin	C21 H20 O11	448.1009	86,588
	Quercitrin	C21 H20 O11	448.1011	456,359
Chol-AL	Betulin	C30 H50 O2	442.3821	22,450
	Jasmonic acid	C12 H18 O3	210.1260	26,781
	Ursolic acid	C30 H48 O3	456.3585	138,092
	Cholest-4-en-3-one	C27 H44 O	384.33955	72,345
Chlo-AF	18-β-Glycyrrhetinic acid	C30 H46 O4	470.34059	3,170,860
	Betulin	C30 H50 O2	442.38204	210,319
	Cholest-4-en-3-one	C27 H44 O	384.33955	866,947
	Quinine	C20 H24 N2 O2	324.18431	839,037
	Tropine	C8 H15 N O	141.11563	415,037
	Ursolic acid	C30 H48 O3	456.36119	847,971
Dw-AF	Ellagic acid	C14 H6 O8	302.0064	110,506
	18-β-Glycyrrhetinic acid	C30 H46 O4	470.3404	402,194
Etac-AF	Andrographolide	C20 H30 O5	350.20763	687,431
	Betulin	C30 H50 O2	442.38136	365,769
	Gallic acid	C7 H6 O5	170.02093	2,488,059
	Ellagic acid	C14 H6 O8	302.00689	1,498,877
	Cholest-4-en-3-one	C27 H44 O	384.33992	998,095
	4-Coumaric acid	C9 H8 O3	164.04681	208,920
	18-β-Glycyrrhetinic acid	C30 H46 O4	470.34053	4,737,974
Hex-AF	18-β-Glycyrrhetinic acid	C30 H46 O4	470.34035	2,623,851
	Cholest-4-en-3-one	C27 H44 O	384.33971	1,979,479
	Betulin	C30 H50 O2	442.38158	854,009
	Ursolic acid	C30 H48 O3	456.35863	317,453
Meoh-AF	Tropine	C8 H15 N O	141.11571	94,846
	Tropine	C8 H15 N O	141.1156	1,710,697
	Rutin	C27 H30 O16	610.1525	203,795
	Quercitrin	C21 H20 O11	448.1011	196,742
	Gallic acid	C7 H6 O5	170.0208	4,709,294
Chlo-AF	Ellagic acid	C14 H6 O8	302.0067	1,877,676
	Cholest-4-en-3-one	C27 H44 O	384.3394	272,430
	18-β-Glycyrrhetinic acid	C30 H46 O4	470.3400	2,093,683
Etoh-AF	Tropine	C8 H15 N O	141.1157	3,376,405
	Quercitrin	C21 H20 O11	448.1014	95,564
	Jasmonic acid	C12 H18 O3	210.1261	107,683
	Gallic acid	C7 H6 O5	170.0208	4,977,273
	Ellagic acid	C14 H6 O8	302.0068	1,895,607
	Deoxycorticosterone 21-glucoside	C27 H40 O8	492.2716	76,108
	Betulin	C30 H50 O2	442.3813	116,072
	Andrographolide	C20 H30 O5	350.2076	744,258
	4-Coumaric acid	C9 H8 O3	164.0467	109,470
	18-β-Glycyrrhetinic acid	C30 H46 O4	470.3404	3,024,916
Dw-AF	4-Methoxycinnamic acid	C10 H10 O3	160.0527	150,602
	Quinine	C20 H24 N2 O2	324.1839	661,869
Acet-AF	16-Hydroxyhexadecanoic acid	C16 H32 O3	272.2341	58,938
	18-β-Glycyrrhetinic acid	C30 H46 O4	470.3402	3,020,794
	3-Coumaric acid	C9 H8 O3	164.0466	175,761
	Ellagic acid	C14 H6 O8	302.0066	4,409,279
	Gallic acid	C7 H6 O5	170.0207	5,845,458
	Quercitrin	C21 H20 O11	448.1010	219,912
	Rutin	C27 H30 O16	610.1542	79,334
	Vitexin	C21 H20 O10	432.1063	192,322

### The correlation matrix of DNA nicking inhibition activity of solvent extracts of *T. arjuna*

Densiometric analysis showed a highly significant (*p* < 0.01) positive correlation (Table 5). The highest correlation with RQ (0.818) is found in RF, followed by band percent (0.755) and lane percent (0.564). RQ had the strongest relationship with band (%), 0.819, followed by RF (0.818) and lane (%), 0.628). Band (%) had the strongest relationship with lane (%) at 0.897, and lane (%) had the strongest relationship with band (%) at 0.897.

**Table 5:** Non-parametric relationship of densiometric value of DNA nicking inhibition potential of solvent extracts of *T. arjuna.*

	Rf	RQ	Band (%)	Lane (%)
Rf	1	0.818**	0.755**	0.564**
*p* value		0.001	0.001	0.003
RQ	0.818**	1	0.819**	0.628**
*p* value	0.001		0.001	0.001
Band (%)	0.755**	0.819**	1	0.897**
*p* value	0.001	0.001		0.001
Lane (%)	0.564**	0.628**	0.897**	1
*p* value	0.003	0.001	0.001	

## Discussion

The present study's qualitative preliminary phytochemical analysis revealed that solvent extracts of *T. arjuna* fruit, bark, and leaf contain tannin and phenolics, steroids, alkaloids, flavonoids, and saponin, which are consistent with previous studies^12^. Tannin and phenolics content was found to be higher in Etoh-AB and Meoh-AB. Etoh-AL, Meoh-AL, Meoh-AF, Etoh-AF, Acet-AB, and Acet-AL, which replaced the earlier findings of^19^, demonstrated either reaction against small solvent extracts or less reaction responses. The qualitative phytochemical screening, in vitro antioxidant operation, and liquid chromatography coupled to electrospray-Orbitrapmass spectrometry analyses all complement and confirm the results. Gallic acid and ellagic acids were present in different proportions, leading to antioxidant and DNA nicking inhibition activity. In a solvent extract, the corresponding region of a specific metabiolite revealed its relative concentration or abundance. Gallic acid, also known as 3,4,5-trihydroxybenzoic acid, is a hydrolysable tannin. Ellagic acid is a dimer of gallic acid formed by oxidative aromatic coupling dimerization. It functions as an antioxidant and can be found in a variety of fruits and leaf extracts. Steroid content was higher in Etoh-AB, Etoh-AF, Meoh-AB, Meoh-AF Acet-AF, Hex-AF, and Etac-AF, which is notable since no studies have highlighted the higher steroid content in *T. arjuna* Hex-AF. Leaf fractions of acetone and methanol contain more saponin than Etoh-AB, Etoh-AF, and Etoh-AL, which is partially consistent with Kumar et al.^20^, who did not report saponin in water fractions. Hex-AF demonstrated a clear presence of alkaloids among all solvent extracts, providing baseline information on this solvent extract, which is consistent with previous studies^21,22^ that indicated that the fruit of the plant contains more alkaloids and can be extracted using adapted techniques with non-polar solvent rather than serial fractions or defatting herbal materials. In comparison to previous findings^23^, which indicated maximum flavonoid content in methanol fraction of *T. arjuna* bark, Etoh-AB showed high flavonoid presence among all solvent extracts, followed by Meoh-AB, Etoh-AF, Acet-AB, and so on. This may be due to variations in processing or processing treatment procedures during extraction. In the current analysis, FRAP, ABTS, NO, and DPPH inhibition (%) was used to estimate the antioxidant capacity of selected solvent extracts of *T. arjuna*, as well as TFC and TPC. The FRAP assay is based on the ability of an antioxidant to reduce a ferric tripyridyltriazine (Fe^3+^-TPTZ) complex to a coloured ferrous tripyridyltriazine (Fe^2+^-TPTZ) complex^23^, which tests the total antioxidant capacity of the material studied. The highest FRAP value was calculated for Etoh-AB, followed by Meoh-AB, Acet-AB, and Dw-AB in bark extracts, and Meoh-AF and Meoh-AL in leaf extracts. Overall, it was greatest in AB, followed by AF and AL. These findings are consistent with those of Kumar et al.^20^ stated that alcoholic fractions have the highest FRAP activities, and bark extracts have higher scavenging activity than leaf extracts, but the quantitative result in this study is substituted due to the higher value of FRAP, indicating the efficacy of the solvent extraction. TPC and TFC follow the same pattern, with Etoh-AB having the highest value, followed by Meoh-AB, Acet-AB, and Dw-AB for fruit extracts, Dw-AF, Acet-AF, and Meoh-AF for leaf extracts, and Etoh-AL having the highest value, followed by Meoh-AL, Chlo-AL, and Dw-AL for leaf extracts. According to the analysis of Jayathilake et al., Etoh-AB had the highest TFC and TPC content among all solvent extracts, which arbitrates the free radical scavenging and strong antioxidant activities. Reference ^24^ proposed a positive, important linear association between antioxidant activity and TPC and TFC contents, suggesting that phenolic compounds and flavonoids were the most abundant antioxidant components in the medicinal herbs studied^2^. According to Fig. 3, Meoh-AB and Etoh-AF have no branching, whereas Etoh-AB and Etoh-AL have the lowest bootstrap value, which may be due to their different responses to antioxidant activities. The current study found that the activities of herbal extracts differ depending on the solvent method used, as well as their response to standard reagents and interactions between solvent extracts. The solvent systems are distributed from right to left based on their polarity and reaction to specifications. Dw-AL connects both parts of the scatter network, indicating that Dw-AL has intermediate activity, and Etoh-AB, Meoh-AB, and Dw-AB are at the extreme left with smaller diameter nodes and connecting edges, indicating their superiority over other solvent extracts, which is consistent with previous studies^19,25^ that highlighted the solvent efficiency and polarity for effectivness. Table 3 shows that DPPH, ABTS, and No inhibition (%) have a positive correlation with FRAP and are strongly correlated with each other, elucidating the antioxidant mediated free radical scavenging ability of the solvent extracts, while TFC and TPC are highly correlated with each other and have a substantial positive correlation with NO >ABTS > DPPH > FRAP. Similarly, from Fig. 4, it can be explained that FRAP, DPPH, ABTS, and NO (%) inhibition is positively correlated with Etoh-AB, Meoh-AB, Acet-AB, and Meoh-AL, while TFC and TPC are positively correlated with Etoh-AL, Dw-AF, Meoh-AF, Etoh-AF, and Etoh-AF, respectively, which is consistent with previous studies^24–28^. Antioxidants have antioxidant properties that protect DNA from oxidative damage caused by reactive oxygen species (ROS), which causes structural changes in the three-dimensional structure of DNA. Furthermore, changes in DNA confirmation impact DNA mobility in an electric field. Despite, the fact that plasmid DNA only showed two bands on an agarose gel, it comes in three distinct types. Form I is a supercoiled circular (relaxed) form that migrates faster than other forms. When the supercoiled DNA form is broken, a nicked circular form (form II) is formed. This form migrates much more slowly than another. Another is form III, a linear form that occurs between forms I and II^29^. Plasmid research looks at the conversion of supercoiled plasmid DNA radicals into linear or circular forms^30^. According to the findings, solvent extracts with DNA nicking inhibition activity result in better RF, RQ, band, and lane efficiency (%). Table 2 shows that Etoh-AB, Meoh-AB, and Acet-AB have the highest activity, followed by Etoh-AF, Acet-AF, Chlo-AL, and Etac-AL. Etac-AF, for example, exhibits reasonably similar effects against plasmid DNA nicking, which is consistent with previous studies^11,30^ that documented the DNA damage prevention activities of non-polar protic solvents as well, as well as a range of antioxidants present in *T. arjuna* extracts. Table 5 shows that RF, RQ, lane (%), and band (%) are highly positively correlated to each other, especially RF with RQ and lane (%) with band (%), which may be due to the involvement of these parameters in DNA nicking recovery of linear form of plasmid DNA, which is consistent with previous studies^31,32^ that have reported that the super coiled circular form of DNA migrates faster than other froms. The results of the bibliographic search revealed a substantial increase in research related to the DNA damage prevention activities of the *T. arjuna* plant, but articles primarily provided densiometric analyses of the DNA nicking inhibition of *T. arjuna* solvent extracts. DNA damage prevention is regarded as a feature of third line antioxidant defences mediated by the presence of TPC and TFC, which supports the current study results indicating solvent extract having more TFC and TPC encompassing more antioxidant potential thereby proving better free radical scavenging potential congruent with all parameters of the study, which is consistent with previous findings^33,34^.

## Material and methods

### Sample collection of plant material and pretreatment of samples

*T. arjuna* bark, leaf, and fruit were extracted with seven different solvents, including hexane, ethyl acetate, chloroform, acetone, ethanol, methanol, and distilled water. Thus, for the analysis, a total of 21 solvent extracts of *T. arjuna* were used. The samples were taken from *T. arjuna* plants on the ICAR-CIFRI campus in Barrackpore, Kolkata, India. Following collection, samples were thoroughly washed under tap water to remove extraneous dust and other materials and allowed to dry overnight at 60 °C in a hot air oven. After ensuring full drying, the sample was ground in a Philips blender and sieved through a 500 micron sieve to extract coarse particles before being filled in airtight polythene bags and stored in a sample jar.

### Preparation of solvent extract

Three parts of *T. arjuna* dry powder were placed in a 1 l capacity conical flask in a 1:5 sample:solvent ratio and held in a shaking incubator at room temperature for 36 h. Following that, the solvent-sample mixture was centrifuged at 6000 rpm for 5 min, and the supernatant was collected and filtered through 110 mm Whatman filter paper and then 90 mm (Whatman no. 1 (40)) filter paper. The residual of the filtration tube and filter paper was put in an aluminium tray and allowed to dry before adding the polar solvent. The filtrate was collected in a beaker and dried under a rotary vacuum evaporator just below the boiling point of the solvent until 1/10th of the original solvent extracts were obtained, which were then stored in amber glass sample vials with holed covers in dark locations. After drying, the solvent extracts were stored in a deep freezer at 4 °C before further use. Both solvent systems were treated in the same way. Fixed volume methods were used for all solvent extracts in in vitro antioxidant studies. In brief, a total of eight different stock concentrations were taken: 25, 50, 75, 250, 500, 750, 1000, and 1250 µg/mL, and 20 µL of each concentration was applied to prepare the sample mixture for analysis.

### Qualitative screening of phytochemicals

For qualitative phytochemical analysis, 1 g dry extracts in 100 mL were dissolved in their respective mother solvents to make a 1% stock concentration, and a reaction was set up to determine essential phytochemicals such as phenols, flavonoids, tannin, saponin, alkaloids, and phytosteroids according to standard procedures^35,36^.

### In vitro antioxidant activity

#### Total phenolic compounds

The Folin–Ciocalteu reagent (1:4 dilutions with distilled water) system was used with minor modifications to estimate total phenolic contents (TPC)^37^. In brief, 10 µL of each concentration was taken from the stocks of 25–1250 µg/mL extracts and combined with 1.5 mL of Folin–Ciocalteu reagent and 5.5 mL of triple distilled water in triplicate test tubes labelled as concentration. The spectrophometer was calibrated at zero with a blank and a control. In the blank, every constituent was present except for the sample, which was replaced with distilled water. The standard was similarly set up in the std labelled test tube, except for the sample. After incubating the reaction mixture at room temperature for 30 min, 1.0 mL of 1 M sodium carbonate was added. The reaction mixture was incubated in a water bath at 40 °C for 20 min before being allowed to cool. In a UV–Vis spectrophotometer, absorbance was measured at 760 nm (EpochTM2 Microplate Spectrophotometer, Biotek, USA). To estimate the TPC of solvent extracts (25—1250 µg/mL), gallic acid (1 mg/mL) was used as a reference. The results were given in µg/mL Gallic acid equivalents (GAE).

#### Estimation of total flavonoids contents

The total flavonoid content (µg/mL) was calculated using a modified aluminium chloride (AlCl_3_) method^38^. 10 L of each concentration (25–1250 µg/mL of extracts) was taken from the stock concentrations and placed in triplicate in a test tube labelled concentration, which was filled with 200 µL distilled water and 150 µL NaNO_2_ and held for 10 min at room temperature in a dark spot. After 10 min, 200 µL (10% AlCl_3_) was added and kept at room temperature in a dark position for another 10 min. Following that, 2 mL (4% NaOH) was applied, and the amount was increased to 5 mL by adding distilled water, and the mixture was incubated for 20 min at room temperature in a dark location. After 20 min, the pink colour produced indicated the presence of flavonoids in the samples, and the OD was measured in a spectrophotometer at 510 nm. To estimate the total flavonoids content of the samples, Quercetin (1 mg/mL) was used as a norm, and TFC was expressed as µg/mL Quercetin equivalents (QE).

#### DPPH free radical scavenging assay

The free radical scavenging behaviour of the solvent extracts was calculated using the 1,1-diphenyl-2-picrylhydrazyl (DPPH) assay, which was slightly changed from^39^. 10 µL of each stock concentration (25–1250 µg/mL of extracts) was taken into triplicate of test tube marked as concentration, then 1 mL freshly prepared DPPH reagent (1 mg/mL in methanol) was applied, and finally, volume was made up 5 mL with distilled water. The control was taken without any samples. The test tubes were incubated at room temperature for 30 min in a dark position before measuring absorbance at 536 nm. The percentage inhibition was determined using the formula below.

$${\text{Inhibition }}\left( \% \right) = \left( {{\text{A}}0 - {\text{A1}}/{\text{A}}0} \right) \times {1}00$$

A0 is the absorbance of control reaction mixture; A1 is the absorbance of sample.

#### ABTS assay

The free radical scavenging activity was calculated using the 2,2′-azino-bis(3-ethylbenzothiazoline-6-sulphonic acid), ABTS radical cation decolorization assay, which was changed slightly from the standard method^40^. In brief, 10 µL of each stock concentration (25–1250 µg/mL of extracts) was taken into triplicate of test tube marked as concentration, to this 3 mL (ABTS prepared with 1:1 of 2.45 mM potassium persulfate, stored in the dark at room temperature for 12–16 h before use, and volume was made up 5 mL adding methanol and adjusting OD 0.734 at 734 nm. The test tube was held at room temperature for 30 min before measuring absorbance at 734 nm. Trolox was used as the norm, and the percentage inhibition was determined using the formula below.

$${\text{Inhibition }}\left( \% \right) = \left( {{\text{A}}0 - {\text{A1}}/{\text{A}}0} \right) \times {1}00$$

A0 is the absorbance of control reaction mixture; A1 is the absorbance of sample.

#### NO scavenging activity

Using the Griess IIIosvoy reaction^41^, the Nitric Oxide scavenging activity was determined. In brief, 10 µL of each stock concentration (25–1250 µg/mL of extracts) was taken into triplicate of test tube marked as concentration, to which sodium nitroprusside (10 mM) in phosphate buffered saline was mixed with different concentrations of extract were dissolved and incubated at 30 °C for 2 h. After that, 10–20 µL freshly prepared Griess reagent (1% sulphanilamide in 2.5% phosphoric acid and 0.1% naphthylethylene diamine dihydrochloride in 2.5% phosphoric acid immediately before use) was applied to the reaction mixture, and the absorbance at 546 nm was calculated after 1 h. Gallic acid acid was used as  standard. The percentage of no scavenging was estimated as follows.

$${\text{Inhibition}} \left( \% \right) \, = \left( {{\text{A}}0 - {\text{A1}}/{\text{A}}0} \right) \times {1}00$$

A0 is the absorbance of control reaction mixture; A1 is the absorbance of sample.

#### FRAP assay

The ferrous reducing antioxidant assay (FRAP) was performed using a slightly changed standard method^42^. From the stock concentrations (25–1250 µg/mL of extracts), 10 µL was taken from each concentration and 1.5 mL FRAP reagent (10:1:1 of 300 mM Sodium Acetate buffer: 20 mM FeCl3: freshly prepared TPTZ in 40 mM HCl), and finally volume was rendered up to 5 mL by adding distilled water. Similarly, a standard (Ascorbic acid) with varying concentrations (25–1250 µg/mL) was set up in triplicate, and a blank without sample or standards was also taken. After that, the reaction mixture was allowed to incubate for 30 min at room temperature in a dark room. At 593 nm, absorbance was measured. The concentration was given in the form of µM (Fe II)/gm of extracts.

### Phytochemical analysis using Liquid chromatography coupled to electrospray-Orbitrapmass spectrometry

3 µL of samples were injected and isolated on a 2.1, 100-mmHypersil gold C18 1.9 micron column (Thermo FisherScientific). With mobile phases A (100% miiliq-water) and B (100% methanol), both containing 0.1% formic acid, the flow rate was 0.3 mL/min. The gradient consisted of an isocratic stage of 2 min at 95% phase A, followed by a linear gradient from 5 to 95% phase before the next 18 min and held for 5 min. The gradient was brought to its initial step in 1 min and kept for 4 min before returning to 100% A for 4 min. An anQ-Exactive plus-Orbitrap hybrid mass spectrometer (Thermo Fisher Scientific) equipped with an electrospray source operating in positive and negative ion modes was used for mass spectrometric detection. The mass spectrometer was set to 4.2 kV capillary voltage and 340 °C capillary temperature. The sheath gas and auxiliary gas flow rates were set to 37 and 13 arbitrary units of nitrogen gas, respectively, with the auxiliary gas heater temperature set to 400 °C. At the MS and MS/MS stages, detection was achieved from 100 to 1200 *m/z* at resolutions of 35,000 and 17,500, respectively. The microscan count was set to one, and the repeat count for dynamic exclusion was set to ten seconds. Thermo Compound Discoverer programme (Version 2.1 SP1 Thermo Fisher Scientific) was used for data analysis, with the workflow Max ID-Detect unknowns with ID using online database searches and the mzCloud search engine.

### DNA nicking inhibition

The plasmid DNA pBR322 (Thermo Scientific) was used to investigate the beneficial effect of *T. arjuna* solvent extracts on hydroxyl radical-mediated DNA damage. First, the extracts were dissolved in 1% tetrahydrofuran (THF) at 50 mg/mL using the fixed dose percentage principle. A reaction mixture (20 µL final volume) of 2.5 µL of 0.25 µg/l plasmid DNA pBR322, 1.5 µL of 1% H2O2, and 16 µL of 50 mg/mL solvent extracts. H2O2 (1%) and tetrahydrofuran (1%) treated plasmid DNAs, tetrahydrofuran (1%) and plasmid DNA and only THF were used as control groups, and the prepared mixture of each solvent extract was incubated at 37 °C for 24 h. 4 µL of bromophenol blue (0.025%) and sucrose (4%) in dH2O loading dye was applied to the mixture and loaded onto the 1% agarose gel. The electrophoresis process was carried out for 45 min at 120 V in the TBE buffer running buffer (pH 8). The Gel was photographed using UV light.

### Statistical tools and soft ware’s for over all association patterns, significance, networking and ordination scaleogram among solvent extracts based on antioxidant potential and gel quantification and characterization

Using the PAST and Minitab 18 softwares, the significance, networking, and ordination scaleogram among solvent extracts based on antioxidant potential was developed after extracting the values for various antioxidant activities across all association patterns. The data was analysed using Microsoft Excel v.16, and significance was determined using SPSS 20. Paint 3D v.16 was used to edit the  images. LAB IMAGE software was used for gel quantification and characterization (Bio-Rad).

## Conclusion

Ethanolic and methanol fruit and bark showed that both antioxidants and free radical scavenging substances were abundant. Results from the phytochemical study showed that polar and polar solvent extracts include the bioactive steroids and other concepts. This preliminary investigation of the extracts of *T. arjuna's* unexplored solvents could provide a starting point for bioactive compounds to design drugs and medicines against geriatric and free radical-caused degenerative disease may lead to an eventual therapeutic intervention.

### Statement of plant material used

The *Terminali arjuna* used in present study was obtained from the plant located in the campus of ICAR-Central Inland Fisheries Research Institute, Barrackpore, Kolkata. For the same Director ICAR-CIFRI is the competent authority who has granted the permission for carry out the study. The tree is big so cannot be stored as specimen, however, it has census authentication number that been also mentioned in our previous research article^43^. Tree has authentication no 14 and plant still exists, it has been used as per the ethical guidelines and not been damaged or killed.

## Supplementary information

Supplementary Informations.
