# Supplementary material for: On valorization of solvent extracts of Terminalia arjuna (arjuna) upon DNA scission and free radical scavenging improves coupling responses and cognitive functions under in vitro conditions
Source: Sci Rep. 2021 May 20;11:10656. doi: 10.1038/s41598-021-88710-w (PMC8137696; doi:10.1038/s41598-021-88710-w)
Supplement: Supplementary file 1 — Supplementary Informations. [file 41598_2021_88710_MOESM1_ESM.doc]

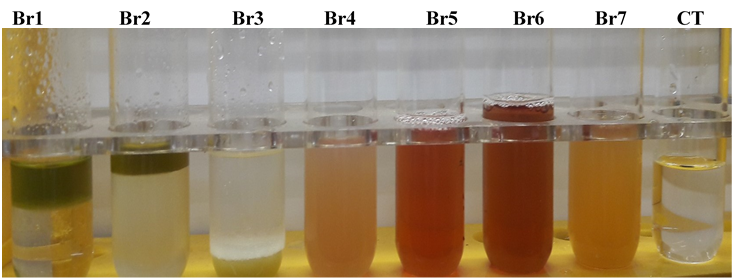

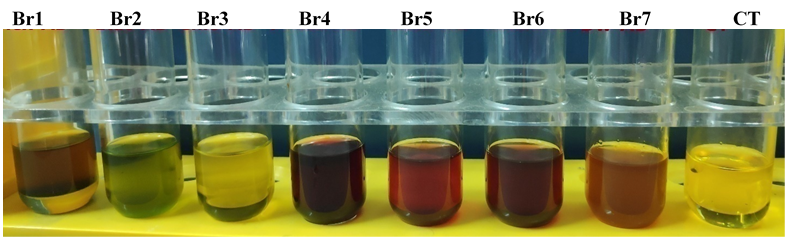

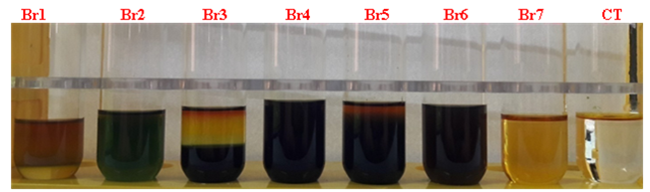

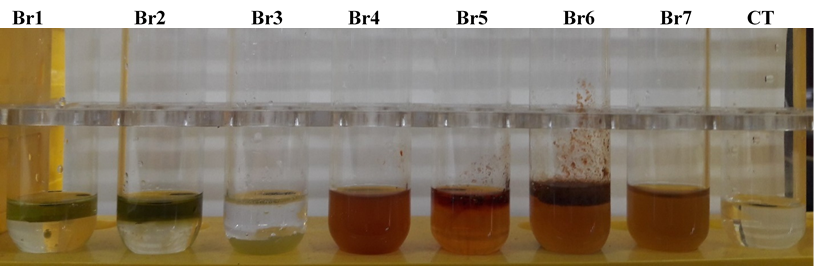

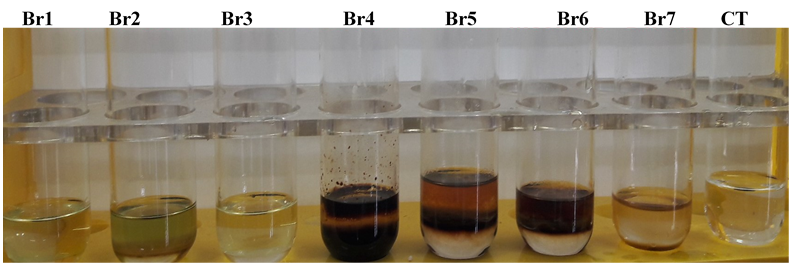


Plate 1: Showing qualitative phytochemical profiling of *Terminalia arjuna* bark extracts. A- Saponin, B-Alkaloid, C- Flavonoid, D- Steroid, E- Tannin


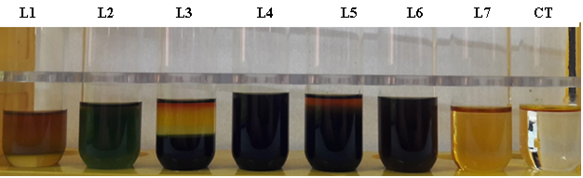

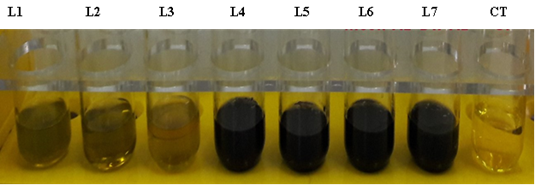

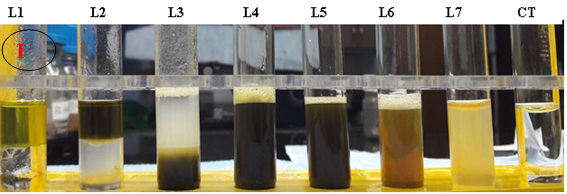

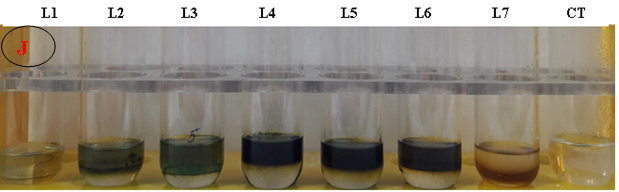

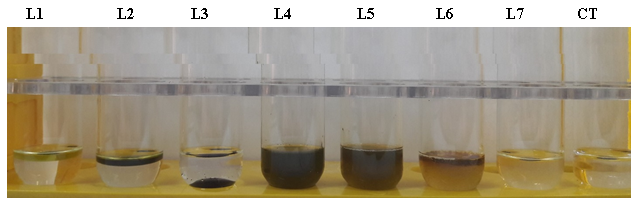


Plate 2: Showing qualitative phytochemical profiling of *Terminalia arjuna* Leaf extracts. F-Alkaloid, G-Flavonoid H-Saponin, I-Steroid, J-Tannin


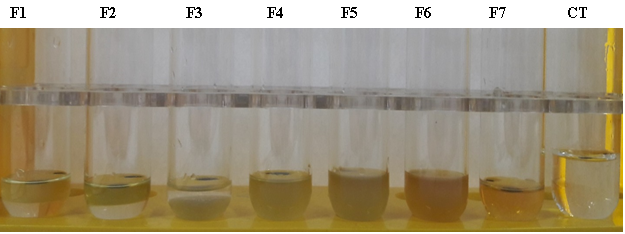

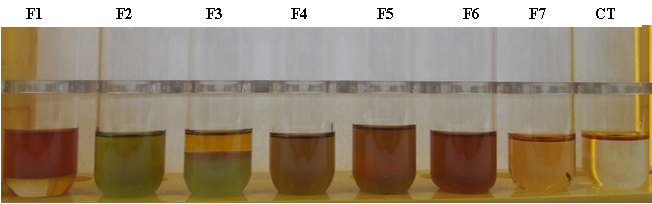

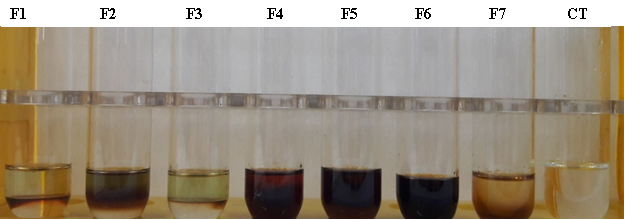

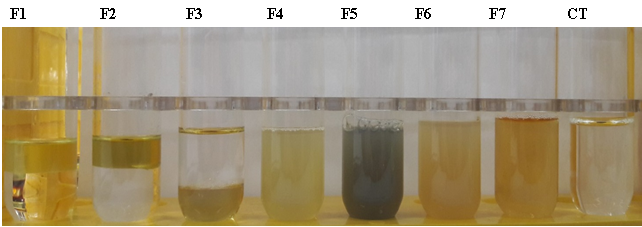

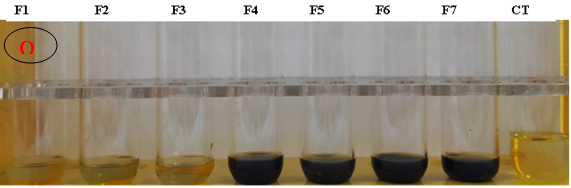


Plate 1: Showing qualitative phytochemical profiling of *Terminalia arjuna* Fruit extracts. A- Saponin, B-Alkaloid, C- Flavonoid, D- Steroid, E- Tanni
